# Effect of coenzyme Q10 on tibial fracture resistance in nicotine-exposed rats

**DOI:** 10.1371/journal.pone.0315462

**Published:** 2025-01-03

**Authors:** Ruan Henrique Delmonica Barra, Bianca Rafaeli Piovezan, Henrique Rinaldi Matheus, Otávio Augusto Pacheco Vitória, Elisa Mara de Abreu Furquim, Luiz Guilherme Fiorin, Ester Oliveira Santos, Juliano Milanezi de Almeida

**Affiliations:** 1 Division of Periodontics, Department of Diagnosis and Surgery, UNESP, São Paulo State University “Júlio de Mesquita Filho”, Araçatuba, Brazil; 2 Nucleus of Study and Research in Periodontics and Implantology (NEPPI), School of Dentistry, Sao Paulo, State University (Unesp), Aracatuba, SP, Brazil; 3 Discipline of Periodontics, School of Dentistry, University of São Paulo, São Paulo, Brazil; Tanta University Faculty of Agriculture, EGYPT

## Abstract

The study aimed to evaluate the potential protection against fractures of oral Q10 supplementation in the tibias of rats exposed to nicotine. Nicotine is known to negatively impact bone density and increase the risk of fractures, in addition to affecting other systems such as the gastrointestinal system, impairing its absorption capacity, negatively affecting bone health. To investigate this, eighty male rats were divided into four groups (n = 20) receiving either nicotine hemisulfate or saline solution (SS) for 28 days. Two daily subcutaneous applications were administered accordingly. Concurrently, vegetable glycerin and Q10 gavage began on day "0". SS: the animals in this group received two daily subcutaneous applications of sodium chloride solution during the entire trial period. 30 days after starting the SS applications subcutaneously, the animals received vegetable glycerin daily until the end of the experiment. SS-Q10: the animals received the SS protocol and daily supplementation with Q10 until the end of the experiment. NIC: The animals received the protocol for NIC and vegetable glycerin daily until the end of the experiment. NIC-Q10: The animals received the protocol for NIC and daily supplementation and Q10 until the end of the experiment. Euthanasia occurred at 7 and 28 days after the beginning the gavage. The tibiae collected were processed for morphometric, densitometric, mechanical, and microtomographic (micro-Ct) analysis. A complementary analysis of intestinal changes was performed. The groups that received Q10 showed slightly better results regarding the mechanical resistance and micro-Ct parameters and to intestinal histomorphometry, as compared with groups not supplemented with Q10. Thus, in rats, it can be concluded that coenzyme Q10 exhibited a protective property to the skeletal system and the gastrointestinal tract, even in the presence of nicotine.

## Introduction

Additional to its well-established impact on heart and respiratory diseases, smoking has been recognized for more than 40 years as a harm to the bone [1]. Smoking is a predisposing factor of the development of osteoporosis and delayed bone repair (e.g. fracture repair) [2]. Nicotine (NIC) is one of the many hazardous substances present in tobacco smoke that impacts on general health. The isolate defects of NIC on bone cells and their repair capacity is supported by considerable literature. Interestingly, the cumulative influence of NIC on bone cells and metabolism culminates with reduced bone density [3], possibly a consequence of decreased volume of trabecular bone, increased osteoclast activity, reduced mineral apposition rate, and impaired osteogenesis [4].

Bone neoformation requires the recruitment of multipotent cells capable of proliferating and differentiating into a wide variety of cell types, including osteoblasts, which are involved in osteoclastic differentiation and function through cell-to-cell interaction [5, 6]. Such events occur when the receptor activator of NF-Kappa β ligand (RANKL) activates its receptor activator of NF-Kappa β (RANK), located in the plasma membrane of osteoclasts and pre-osteoclasts, positively regulating bone resorption. On the other hand, a negative regulation of bone resorption occurs when osteoprotegerin (OPG) binds to RANKL, preventing its interaction with RANK and the occurrence of the aforementioned events [7, 8]. It is known that nicotine has a direct effect on proliferation and maturation of osteoblasts [9–11], which may have an indirect effect on bone resorption, via regulation of the RANK/RANKL/OPG system. Tanaka et al. [12] reported that NIC increases the number of osteoclasts. Increased RANKL or decreased local expression of OPG can cause bone resorption in several locations in the human skeleton [13]. The impact of NIC in the intricacy of bone formation/resorption substantiates its harm to bone repair process [14], the higher rate of bone loss, the reduced bone quality and density, thus characterizing NIC as a risk factor for the occurrence of fractures [15–17].

Apart from the direct action on bone tissue, studies have shown that NIC can modify the integrity of the intestinal mucosa, affecting its permeability and, consequently, the absorption of nutrients [18], which is fundamental for the preservation of bone mineral density [19]. The main nutrients involved in bone health include calcium, vitamin D, phosphorus, magnesium and vitamin K, each of which performs specific and interdependent functions fundamental to the formation and maintenance of the bone matrix [20]. The literature also indicates that NIC can interfere with the expression of transporters of some of these nutrients, altering their absorption, such as iron and calcium [21].

Interestingly, the undesirable catabolic effects of nicotine on cells could be reversed by antioxidants, such as Pycnogenol or coenzyme CoQ10 [22]. CoQ10 is a natural co enzyme used for the treatment of various human diseases, such as cardiomyopathies, Parkinson’s, Alzheimer’s, diabetes, dystrophies and periodontal disease [23]. It is a fat-soluble substance with antioxidant properties synthesized in the liver and available in foods or supplements. CoQ10 plays a crucial role in adenosine triphosphate (ATP) production, where it is involved in redox processes and in the transport of electrons in mitochondria, eliminating reactive oxygen species (ROS) and free radicals, protecting cells against oxidative stress and aging [24–26]. These regulatory effects of CoQ10 decrease osteoclastogenesis and increases osteogenesis [24]. When construed together, the overlapping mechanisms between NIC-mediated bone damage and the protective effect of CoQ10 on bone suggest that CoQ10 may be efficient for preventing skeletal complications.

This assumption is plausibly supported by its role in modulating bone health, especially in the context of nicotine-induced oxidative stress. Research shows that CoQ10 can improve mitochondrial function and reduce osteoblast apoptosis by increasing the availability of ATP, which is essential for fundamental metabolic processes [27]. Furthermore, recent evidence suggests that CoQ10 can influence the expression of growth factors, such as vascular endothelial growth factor (VEGF), which plays a key role in angiogenesis and arrangement of a microenvironment conducive to bone regeneration [28]. CoQ10 has also been associated with the regulation of inflammatory pathway signaling, such as the NF-kB pathway, which is directly linked to osteoclastogenesis and bone resorption [29]. In this way, CoQ10 not only acts as an antioxidant but also offers a multifaceted approach that may counteract the deleterious effects of NIC, substantiating its potential as an innovative therapeutic intervention for the mitigation of smoking-related harms.

Thus, the present study aimed to provide new insights by evaluating not only the effects of oral CoQ10 supplementation on skeletal parameters and fracture resistance of rats receiving NIC, but also investigating structural changes in intestinal villi. These investigation may position CoQ10 as a potential therapeutic strategy to mitigate nicotine-induced bone damage and its systemic implications, especially in the prevention of fractures.

## Material and methods

### Animals

**Fig 1:**
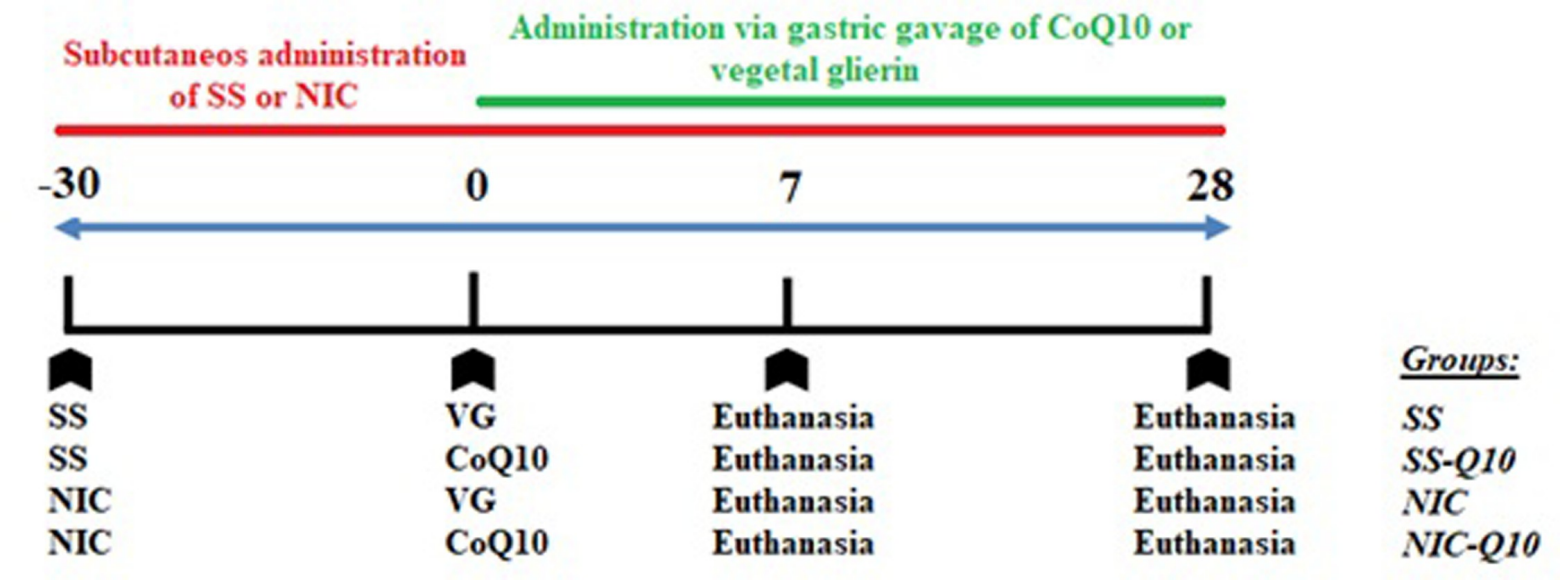
Experimental design. Experiment design timeline showing periods of application of substances, periods of euthanasia and group divisions.

Eighty healthy 3-month-old male Wistar rats (Rattus norvegicus albinus), each weighing 200–250 g, were used following a blind, randomized and controlled design. The animals were housed in cages with 5 animals each, in a temperature-controlled environment (22˚C±1˚C, 70% humidity) with a 12-hour light-dark cycle and ad libitum access to water and food. The study was conducted in accordance with the ARRIVE Guidelines: Animal Research: Reporting in Vivo [30] and have included the ARRIVE checklist. It was conducted at the Department of Diagnosis and Surgery—Division of Periodontics and approved by the Ethics Committee of Animal Use (CEUA) under the protocol #00385–2020, São Paulo State University (UNESP), School of Dentistry, Araçatuba, São Paulo, Brazil. The sample size was calculated to achieve a power of 0.8 and an alpha error of 0.05 based on a potential standard deviation of 12%, and the assumption that a 10% difference between groups/periods would be relevant. Histometric analysis was the primary outcome parameter used to determine sample size. The distribution of groups (n = 20) and the complete design of the experiment can be seen in Fig 1.

For randomization, numbers from 1 to 80 were labeled in the upper tail of animals. A blinded staff external to the study uploaded the number sequence to the software Minitab® 17 (Minitab Inc., State College, PA, USA) to generate a randomization table for allocating (1:1 allocation ratio) the animals to groups.

### Administration protocol

Regarding substances, every 12 hours the animals received subcutaneous injections of nicotine or saline solution in the dorsal region at a pre-established time until the end of the experimental period. Nicotine hemisulfate (Sigma, St. Louis, MO, USA) was diluted in saline at a concentration of 5mg/ml. For each animal was administered a volume of the diluted solution, at a dosage of 3mg/kg of body weight according to the study protocol (Okamoto, Kita, Okuda, Tanaka, & Nakashima 1994) [31]. The animals were weighed weekly to adjust the doses. The administration of saline solution (0.9% sodium chloride) was carried out with the aim of simulate the same conditions of daily stress resulting from nicotine applications.

Coenzyme Q10 was administered by gavage once daily throughout the experiment. The CoQ10 solution was prepared using vegetable glycerin as a vehicle. The concentration of the final product was 120 mg of CoQ10/ml of glycerin [25]. To simulate the stress of gavage with CoQ10, animals in the SS and NIC groups received only vegetable glycerin as an administration protocol. The solution was administered at a dosage of 1 ml with a syringe attached to a stainless-steel gavage needle and introduced through the animal’s oral cavity, applied slowly and carefully so as not to cause damage to the animals.

### Anesthesia

The surgical euthanasia procedures were stated by sedation and general anesthesia, obtained by the combination of xylazine hydrochloride (6 mg/kg of body weight) and ketamine hydrochloride (70 mg/kg of body weight).

### Euthanasia

Animal euthanasia, in each established period, was carried out following strict technical and ethical control. The method used for euthanasia was the chemical method by administering an excessive dose of anesthetic, following the guidelines established by regulatory bodies, such as the National Council for the Control of Animal Experimentation (CONCEA) and the American Veterinary Medical Association (AVMA). All steps for euthanasia were carried out with a protocol to minimize pain, suffering and with the least possible trauma, carrying out humane management, ensuring the use of ethical animal welfare standards. The researcher responsible for this procedure was previously trained and approved by CONCEA in carrying out euthanasia procedures. The euthanasia procedure was performed with a lethal dose (150 mg/kg) of sodium thiopental (Cristália Ltda., Itapira, SP, Brazil), after general anesthesia. Considering that thiopental solution has an alkaline pH and, therefore, may cause irritation or pain, lidocaine (4mg/kg) was added to the solution. The substances were injected intraperitoneally upon prior fasting, to avoid regurgitation and aspiration of gastric contents. All of these procedures were carried out in a calm environment and away from other animals, ensuring minimum stress. To confirm the animal’s death, complementary methods were used to evaluate vital signs, such as checking the absence of cardiac, respiratory, pupillary reflexes and any residual brain activity, which is mandatory to ensure the effectiveness of the procedure.

### Tissue processing and histometric analysis

Conventional histological processing was performed with paraffin embedding to obtain semi-serial sections 4 μm thick, following a longitudinal section plane in relation to the area of interest corresponding to the intestinal villi and crypts of the groups at 28 days (n = 40). Six equidistant sections of each specimen were stained with hematoxylin and eosin (H&E) for histometric analysis, through favorably oriented sections selected from each animal, cut perpendicularly from the villus enterocytes to the muscularis mucosa. The analyses were performed by examiners (NJA and EE) who were calibrated and masked to the treatments performed.

### Morphometry, mechanical and densitometry analysis

The animals were weighed weekly to assess body mass. After euthanasia, the right and left tibias were dissected and weighed on a precision scale with 0.01 g divisions (n = 160). The length of the tibias was measured with a digital pachymeter with 0.01 mm precision. For the mechanical analysis, the left tibias were submitted to three-point flexion tests, with a Universal Testing Machine (EMIC ®, DL 10000 model). The bones were positioned on two support points 25 mm apart. A 200N load cell was used and the force was applied at a speed of 1 mm/min until the sample broke. The analyzed properties were the maximum strength and relative stiffness of each sample. The right tibias were sent for microtomographic analysis using a Microtomography device (FOA/UNESP Multiuser Laboratory–SkyScan 1272) in the following settings: X-ray voltage 70 kV, X-ray current 143 mA, Al filter 0.5 mm, pixels 8 μm, camera resolution setting 2016×1344, tomographic rotation 180°, rotation step 0.5 and scan duration approximately 55 minutes.

### Statistical analysis

Data were analyzed using BioStat software (BioStat version 5.0, Mamirua Institute, Manaus, AM, Brazil). The normality of data distribution was tested using the Shapiro-Wilk test. The data from the analyzes of the intestines, as well as those of the tibias, were tested with Two-way Analysis of Variance (ANOVA) and Tukey’s post-test. The significance level for the tests was p≤0.05.

## Results

Regardless of experimental group, all animals used in this experiment were healthy and no complications were observed during this study.

### Histometric analysis of the intestines

**Fig 2:**
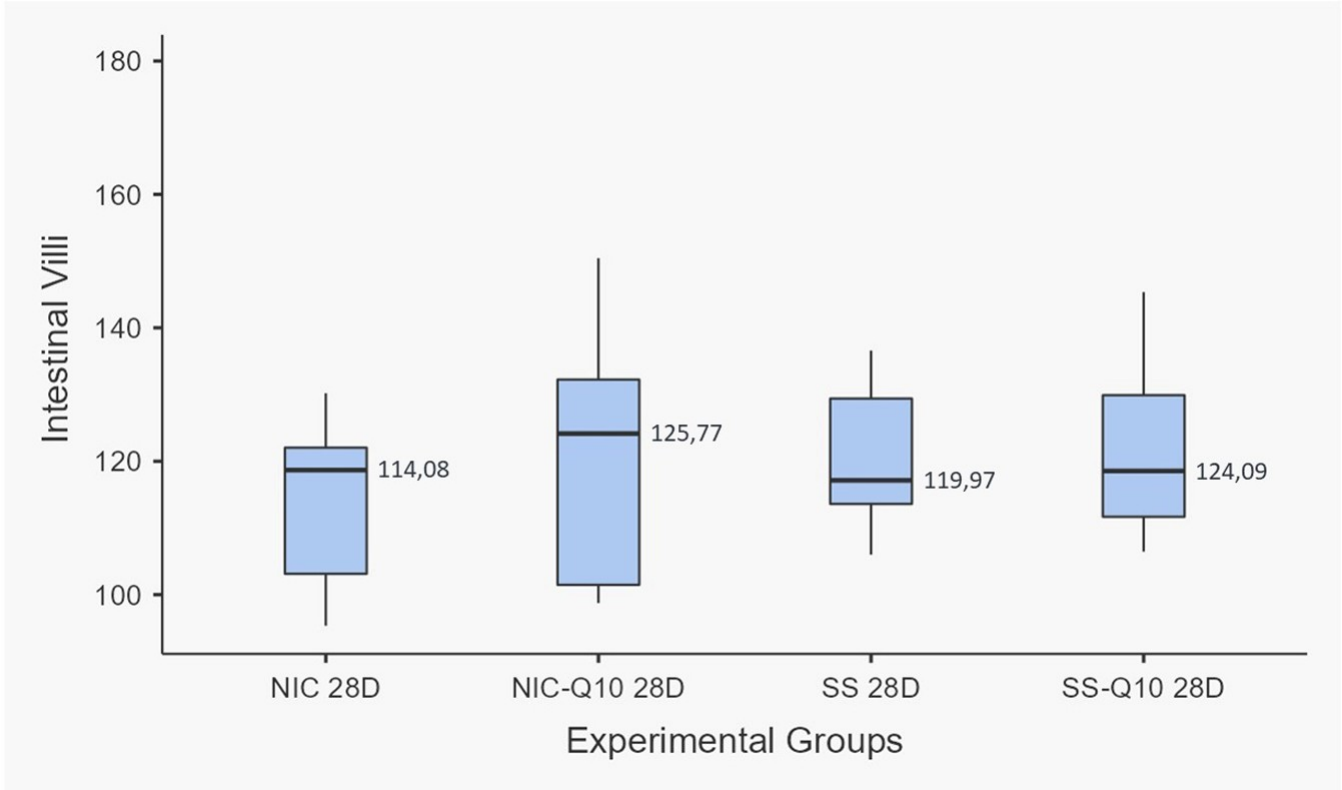
Length of intestinal villi. Graph representing the values (mean ± standard deviation) of intestinal villi length. Source: from the authors themselves.

**Fig 3:**
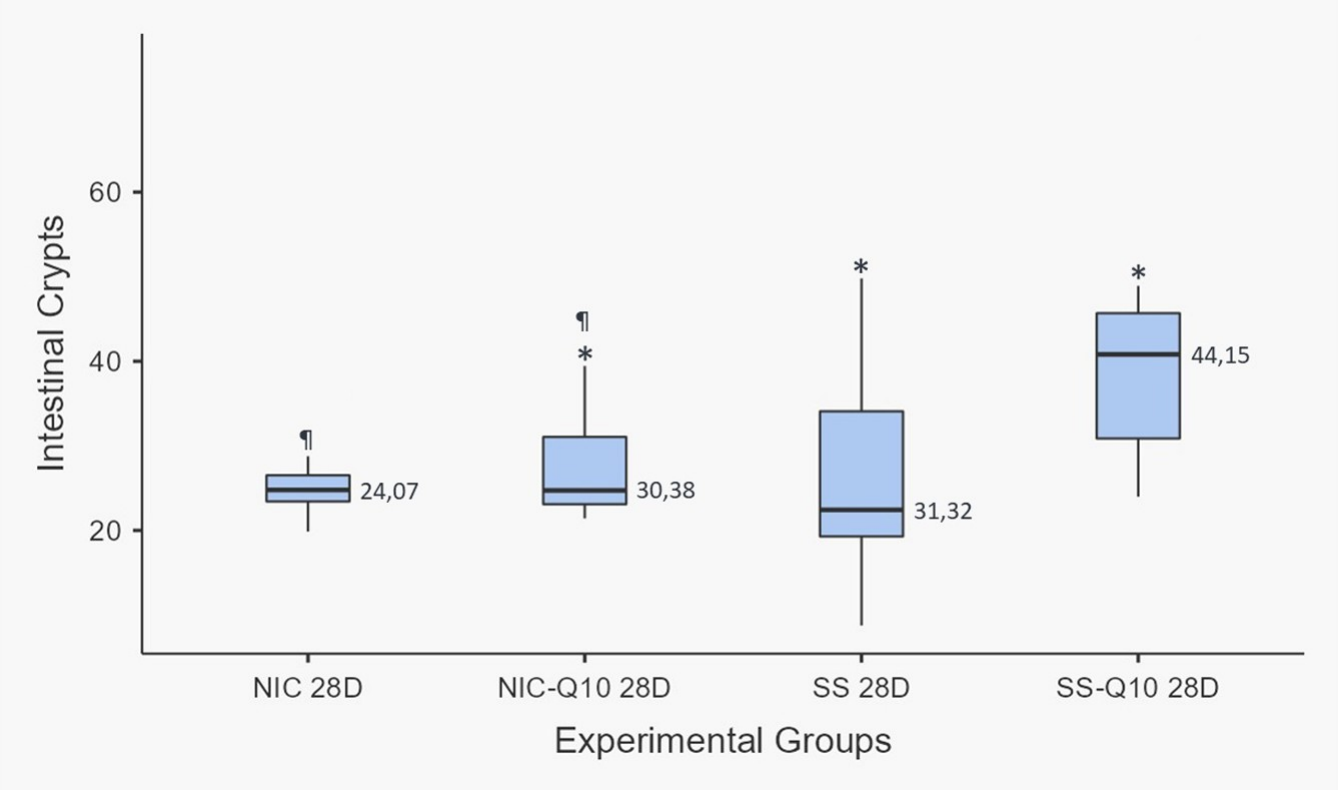
Height of intestinal crypts. Graph representing the values (mean ± standard deviation) with the distribution of individual data on the heights of the intestinal crypts for each group. Caption: (*) statistically significant difference with the NIC group; (¶) statistically significant difference with the SS-Q10 group. Statistical test: Shapiro-Wilk, One-way ANOVA and Tukey test (p≤0.05). Source: from the authors themselves.

**Fig 4:**
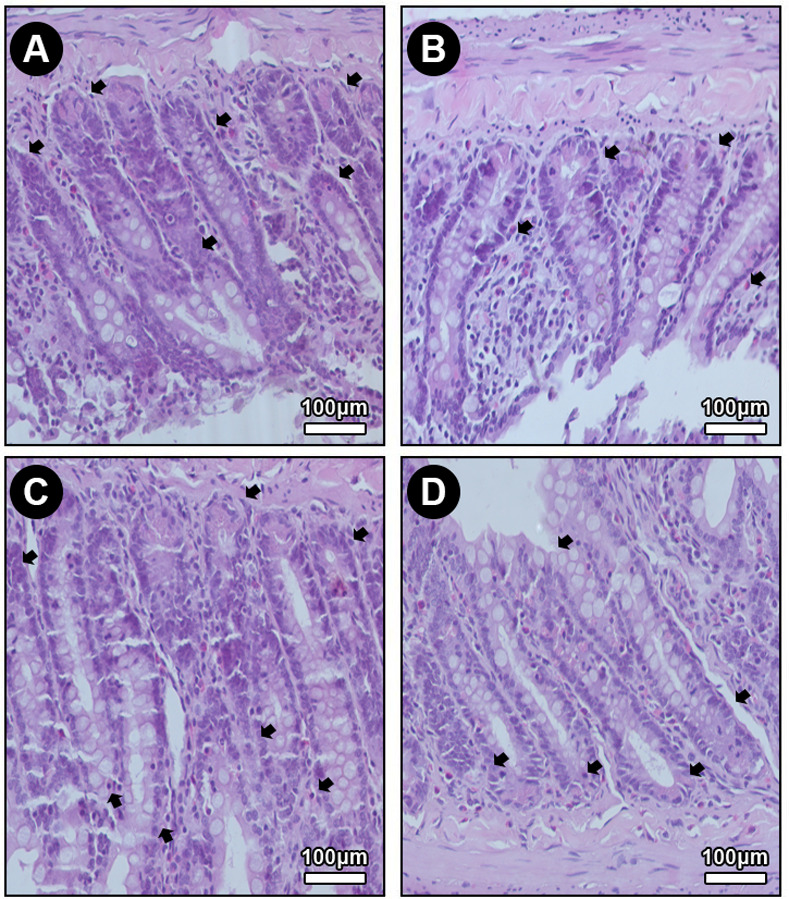
Histology of the intestines. Photomicrographs showing the histological longitudinal section of the colon mucosa in groups SS (A), SS-Q10 (B), NIC (C) and NIC-Q10 (D). In the colon, the villi are absent, with only the tubular glands (crypts), shown by black arrows. Staining: hematoxylin and eosin. Magnification: 40x. Source: from the authors themselves.

Agreement of 97.72% between measurements was identified using Cohen’s Kappa coefficient. Figs 2 and 3 show the results for each group. In the intergroup analysis, the SS, SS-Q10, NIC and NIC-Q10 did not show statistically significant differences when evaluating the length of the villi. When evaluating the height of the intestinal crypts, the NIC group presented a lower height when compared to the other groups and the SS-Q10 group presented a greater crypt height when compared to the NIC-Q10 group (Fig 4). Histologically, the projections of the villi intestinal crypts appear normal in all groups, being differentiated only by the lower height of the intestinal crypts associated with the groups that received nicotine.

### Morphometric analysis of the tibias

**Fig 5:**
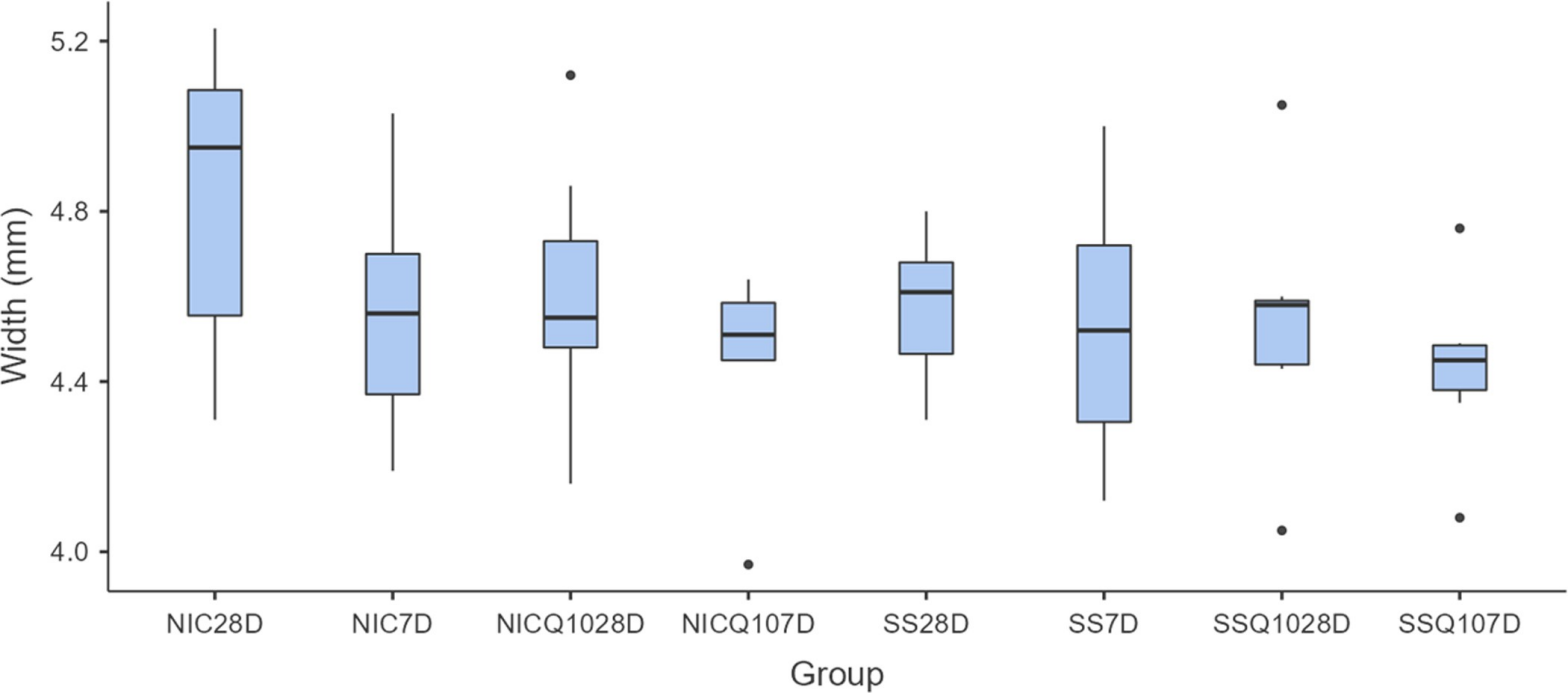
Tibia width. Graph representing the values in mm (mean ± standard deviation) with the distribution of individual data on tibial widths for each group and period. Source: from the authors themselves.

**Fig 6:**
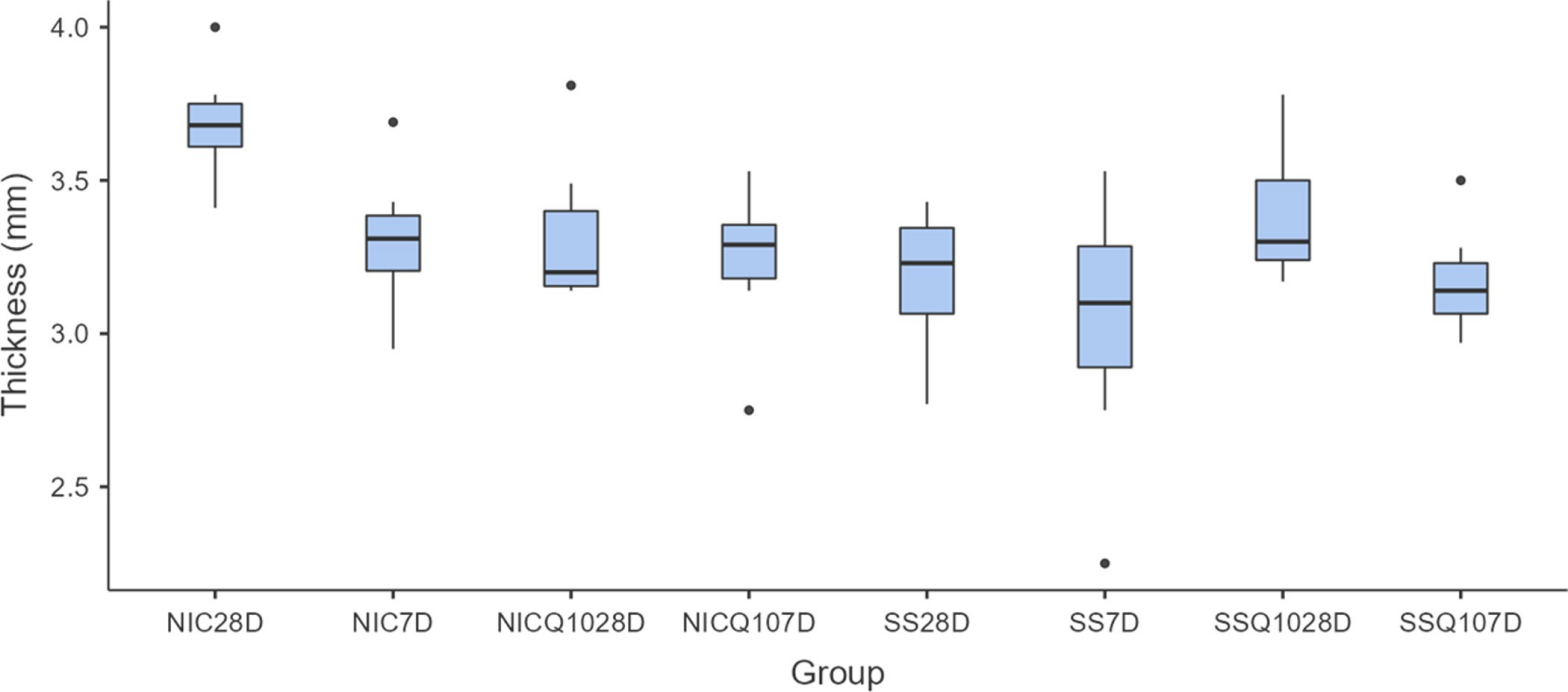
Tibia thickness. Graph representing the values in mm (mean ± standard deviation) with the distribution of individual data on tibial thickness for each group and period. Source: from the authors themselves.

**Fig 7:**
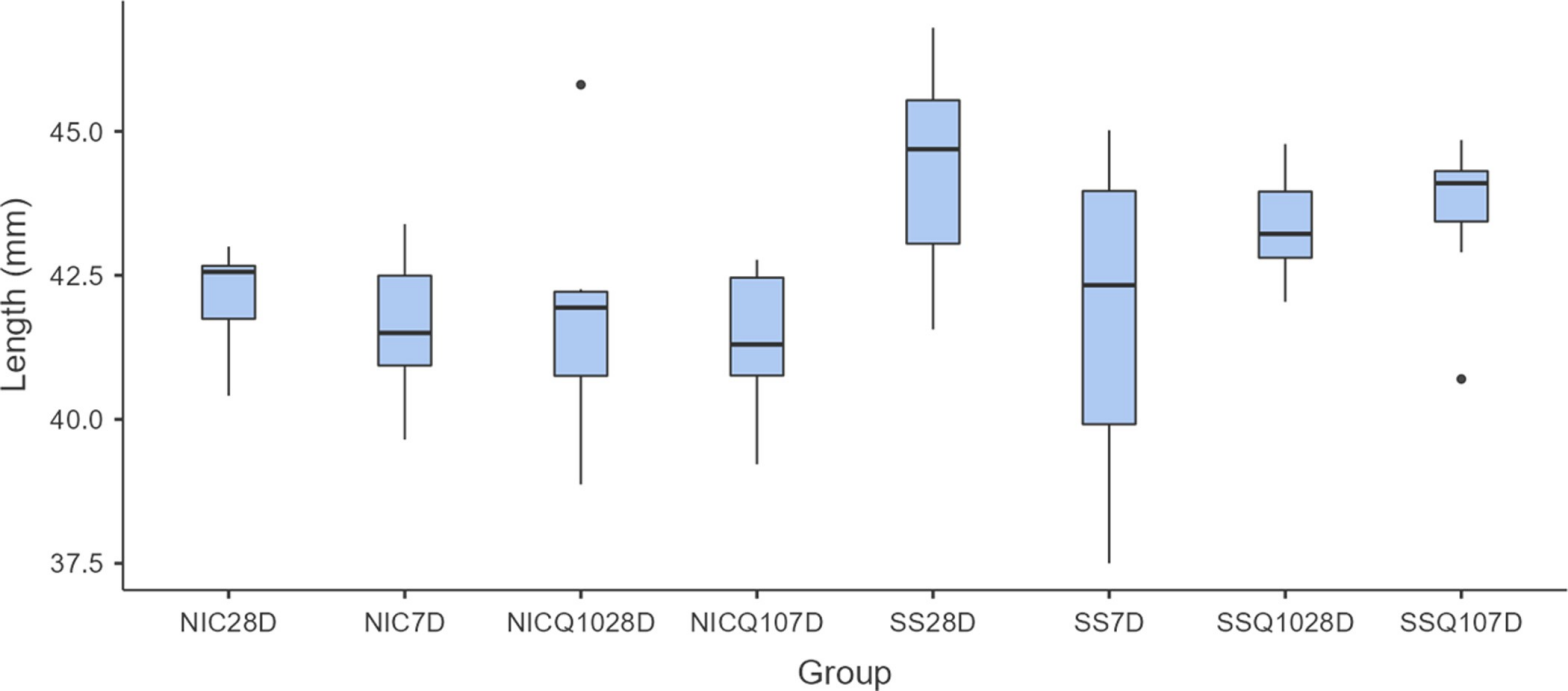
Tibia length. Graph representing the values in mm (mean ± standard deviation) with the distribution of individual data on tibial length for each group and period. Source: from the authors themselves.

**Fig 8:**
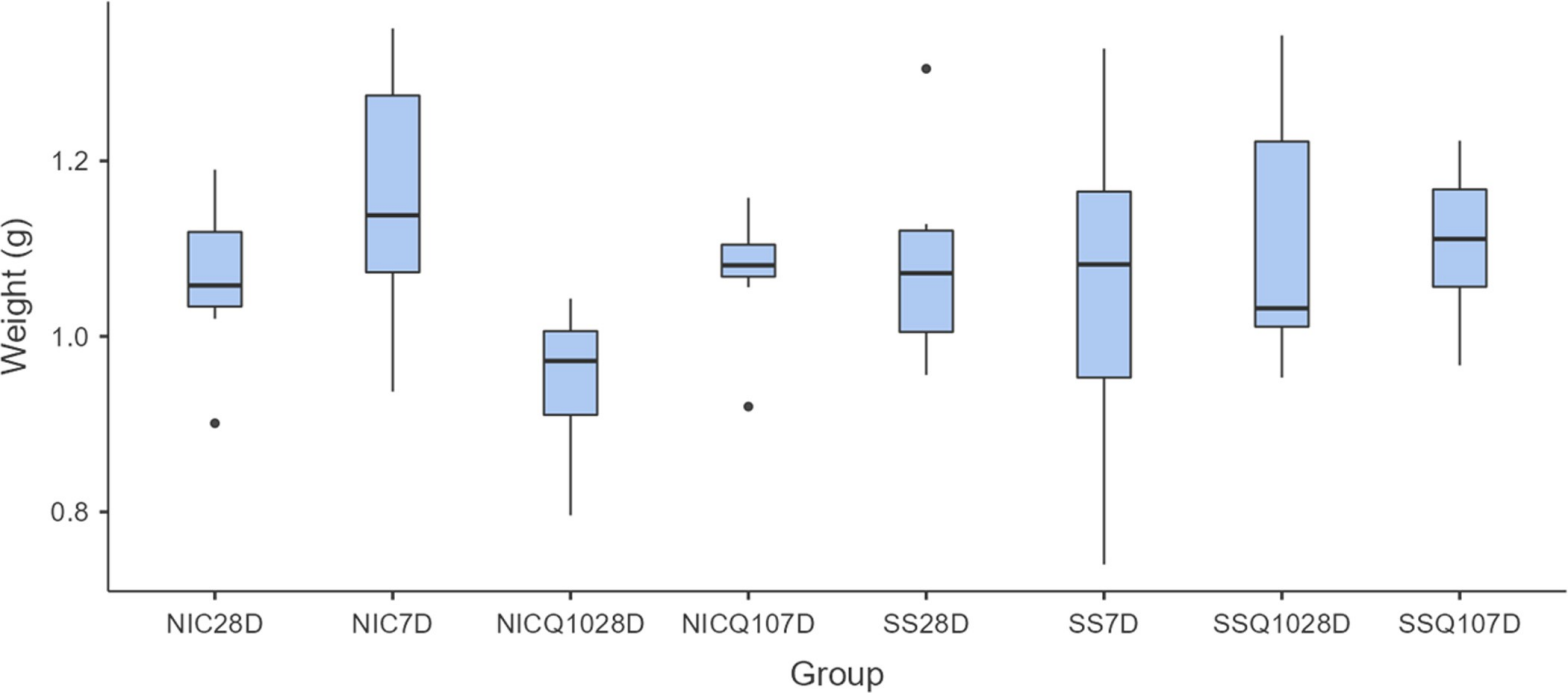
Tibia weight. Graph representing the values in g (mean ± standard deviation) with the distribution of individual data on tibial weight for each group and period. Source: from the authors themselves.

There were no significant statistical differences, regardless of intergroup or intragroup comparisons, in the morphometric analyzes of the tibias. The means and standard deviation of the width, thickness, length and weight of the tibias for each group and period are shown in Figs 5–8.

### Mechanical analysis of the tibias

**Fig 9:**
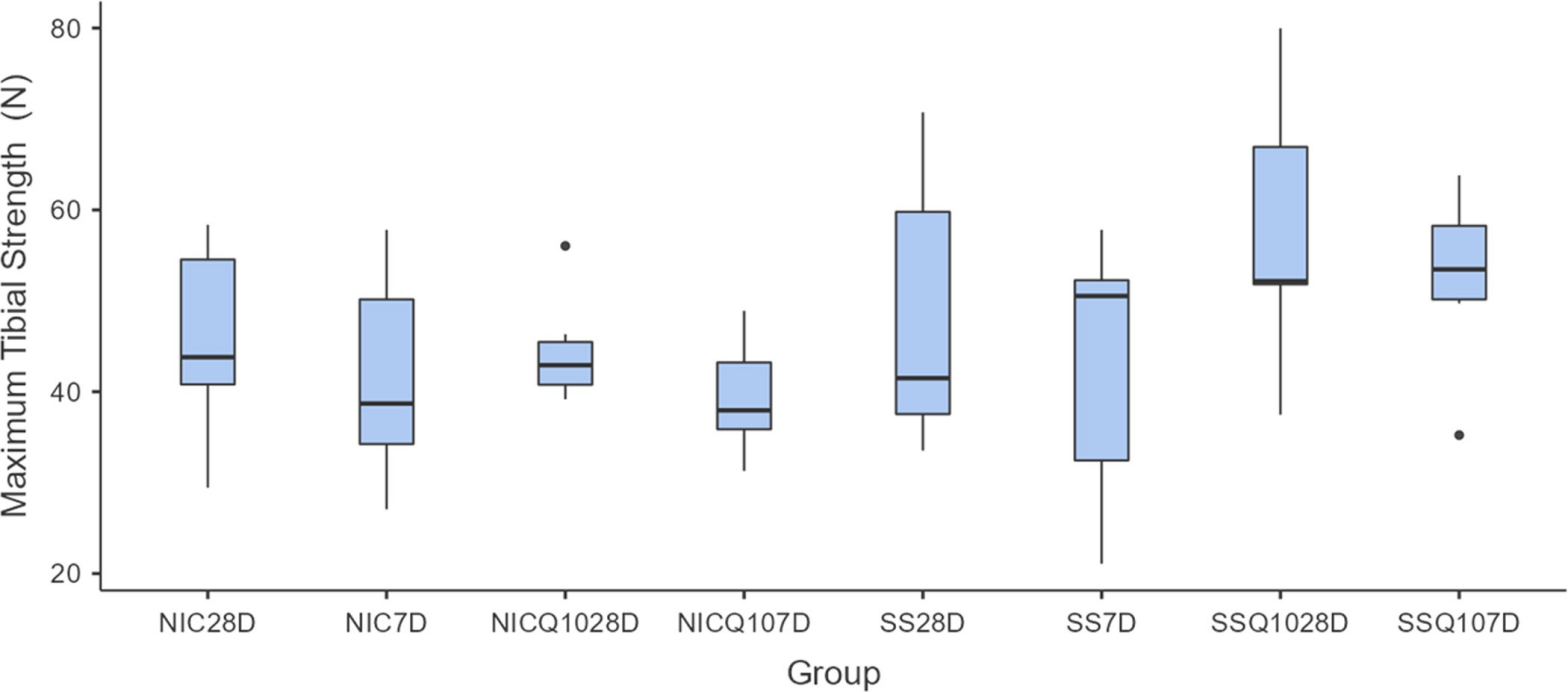
Maximum tibial strength. Graph representing the values in N (mean ± standard deviation) with the distribution of individual data on maximum tibial strength for each group and period. Source: from the authors themselves.

**Fig 10:**
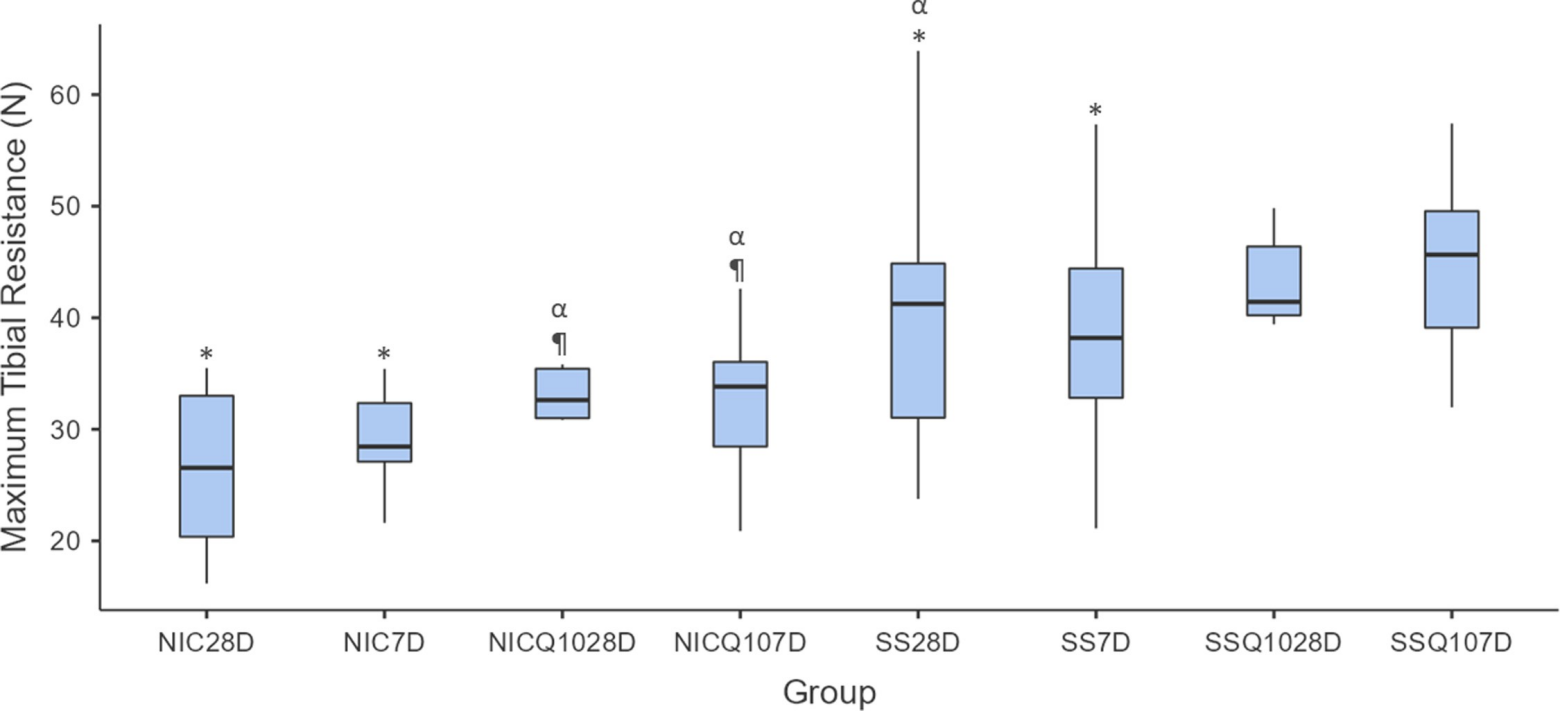
Maximum tibial resistance. Graph representing the values in N (mean ± standard deviation) with the distribution of individual data on maximum tibial resistance for each group and period. Statistical test: Shapiro-Wilk, Two-way ANOVA and Tukey test (p≤0.05). Source: from the authors themselves. Legend: (*) statistically significant difference with the SS-Q10 group in both periods; (¶) statistically significant difference with the SS-Q10 group only at 7 days; (α) statistical difference with the NIC group at 28 days.

**Fig 11:**
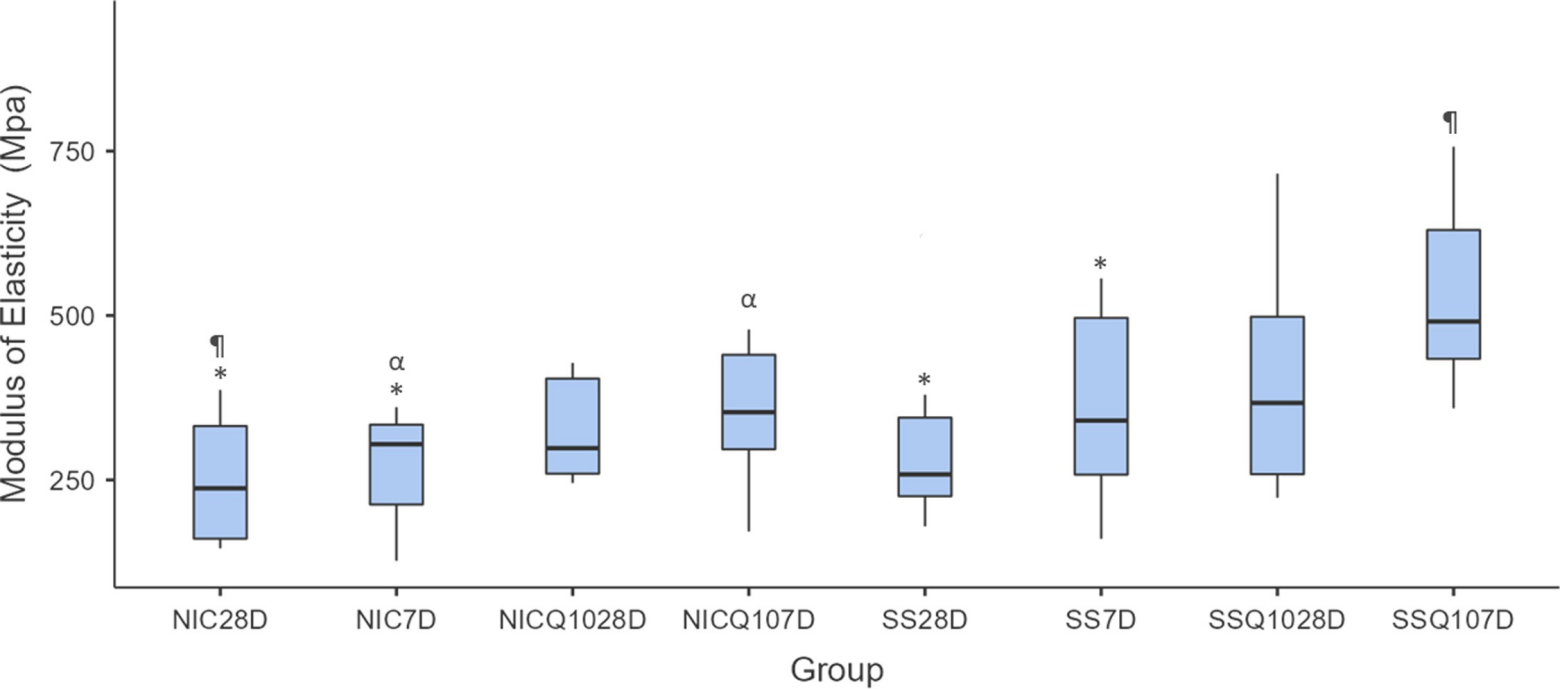
Modulus of elasticity. Graph representing the values in MPa (mean ± standard deviation) with the distribution of individual data of the modulus of elasticity of the tibias for each group and period. Statistical test: Shapiro-Wilk, Two-way ANOVA and Tukey test (p≤0.05). Source: from the authors themselves. Legend: (*) statistically significant difference with the SS-Q10 group in both periods; (¶) statistically significant difference with the NIC-Q10 group in both periods; (α) statistical difference between groups in the same period.

The means and standard deviation of mechanical analysis for each group and period are shown in Figs 9–11. During the intragroup analysis, the SS-Q10 group in both periods showed greater resistance when compared to the SS and NIC. In comparison to the NICQ10 group, in both periods, SS-Q10 showed a statistically significant difference, revealing greater resistance only at 7 days. Finally, the NIC group at 28 days showed lower resistance compared to the NICQ10 group in both periods.

Comparing the modulus of elasticity of the groups, it was possible to observe that the SS-Q10 group had a higher modulus of elasticity when compared to the SS and NIC groups in both periods. The NIC-Q10 group in both periods showed a higher elasticity modulus when compared to the NIC group at 28 days and a lower elasticity modulus when compared to the SS-Q10 group at 7 days. And finally, the NIC group at 7 days showed a higher elastic modulus compared to the NIC-Q10 group in the same period.

When the intergroup and intragroup comparison analyzes were performed, there were no statistically significant differences in the value of maximum strength between groups in both periods.

### Analysis of computerized Microtomography (Micro Ct) and densitometry tibial bone

**Fig 12:**
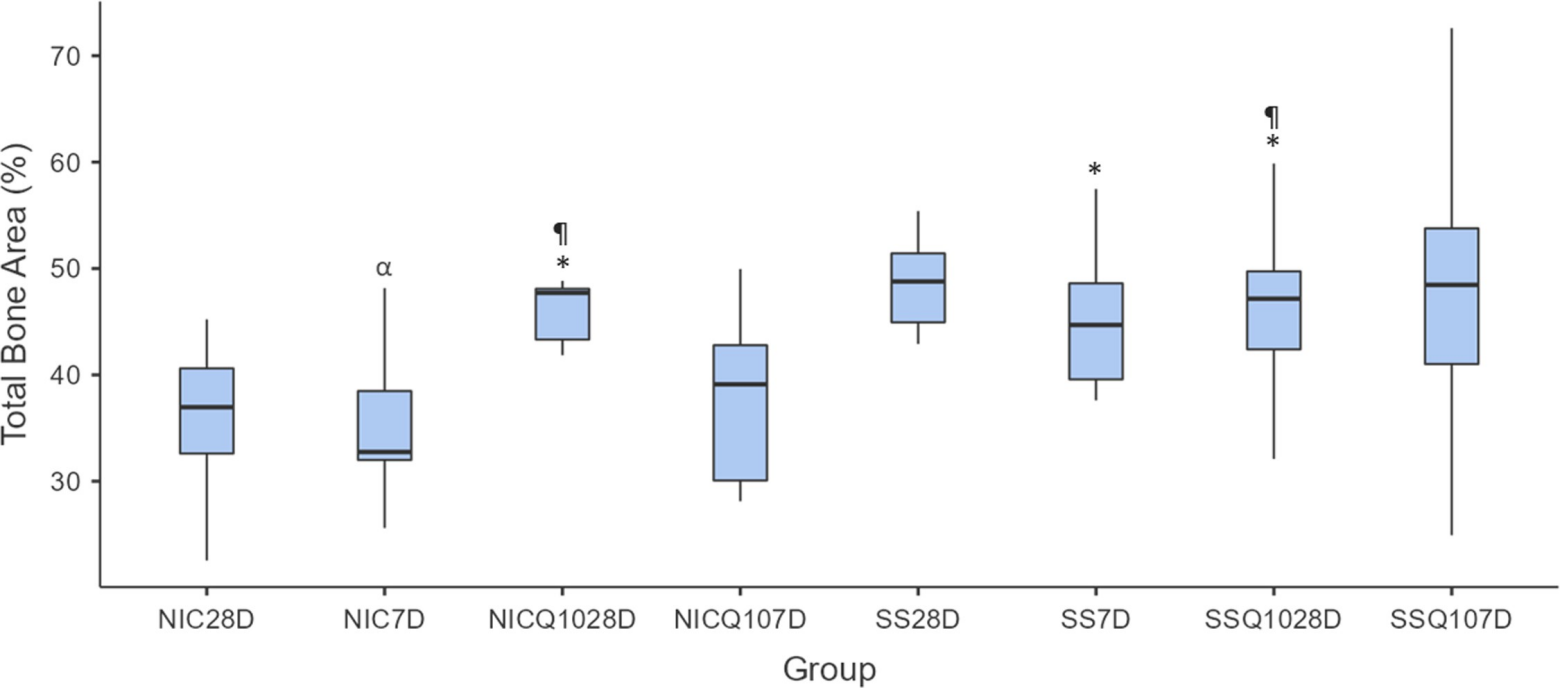
Total bone area. Graph representing the values in % (mean ± standard deviation) with the distribution of individual data of total bone are of the tibias for each group and period. Statistical test: Shapiro-Wilk, Two-way ANOVA and Tukey test (p≤0.05). Source: from the authors themselves. Legend: (*) statistically significant difference with the NIC group at 7 days; (¶) statistically significant difference with the NIC group at 28 days; (α) statistically significant difference with the NIC-Q10 group at 28 days.

**Fig 13:**
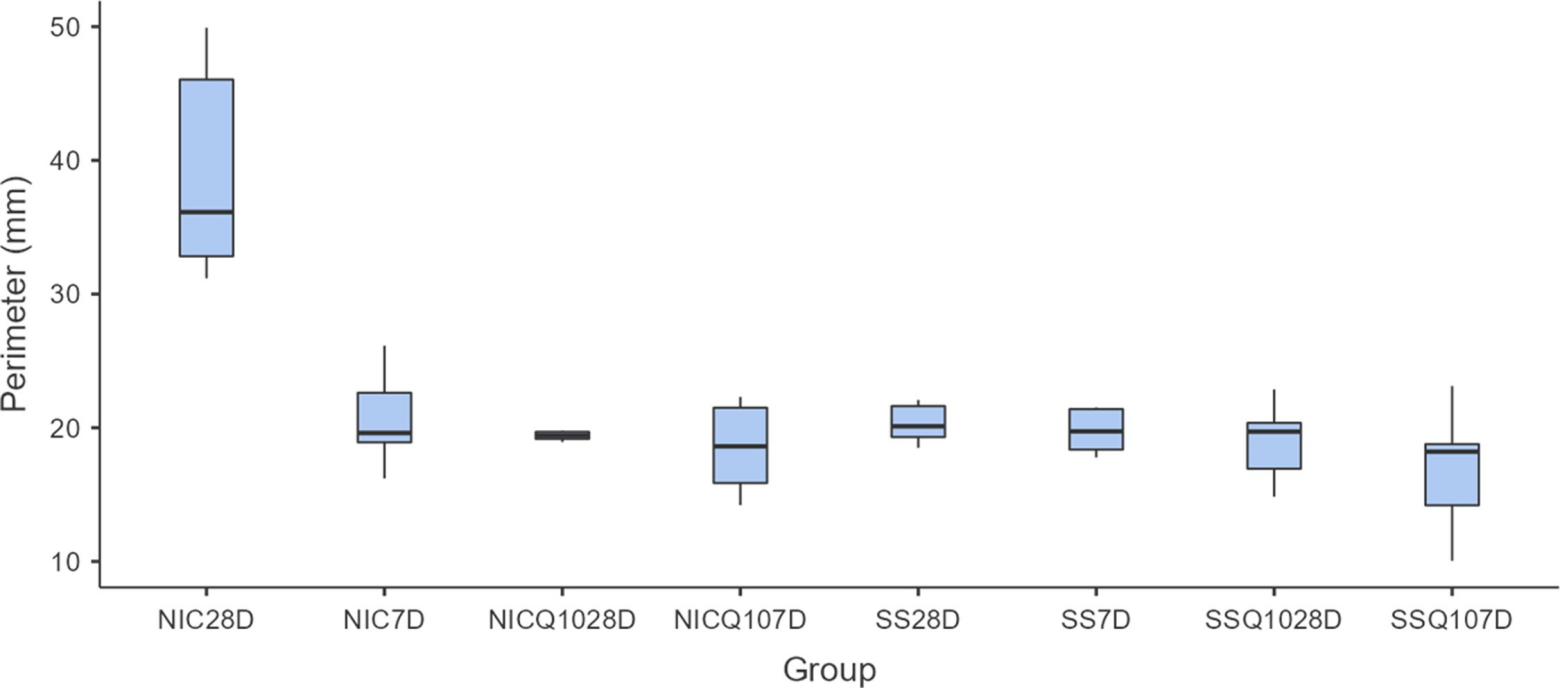
Tibia perimeter. Graph representing the values in mm (mean ± standard deviation) with the distribution of individual tibia perimeter data for each group and period. Source: the authors themselves.

**Fig 14:**
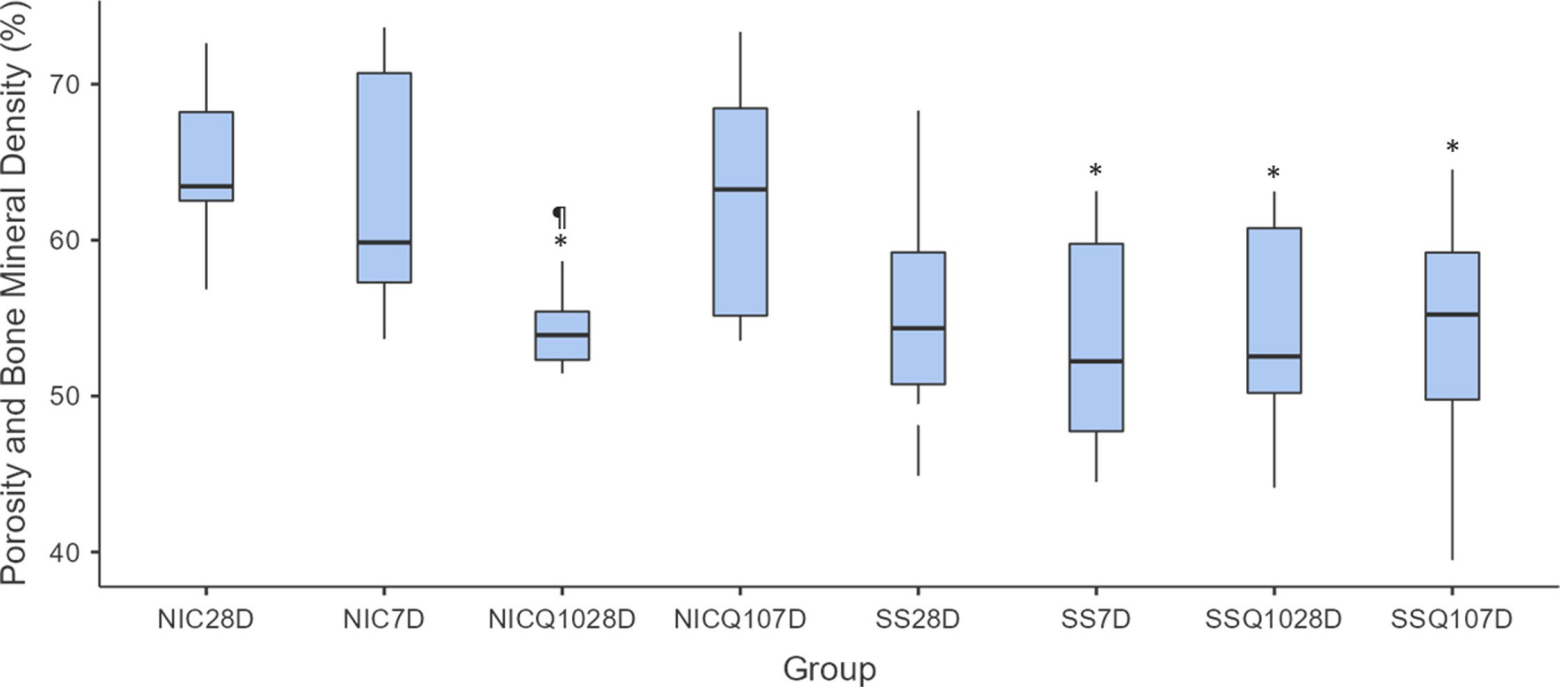
Porosity and bone mineral density. Graph representing the values in % (mean ± standard deviation) with the distribution of individual data of porosity and bone mineral density of the tibias for each group and period. Statistical test: Shapiro-Wilk, Two-way ANOVA and Tukey test (p≤0.05). Source: from the authors themselves. Legend: (*) statistically significant difference with the NIC group in both periods; (¶) statistically significant difference with the NIC-Q10 group at 7 days.

**Fig 15:**
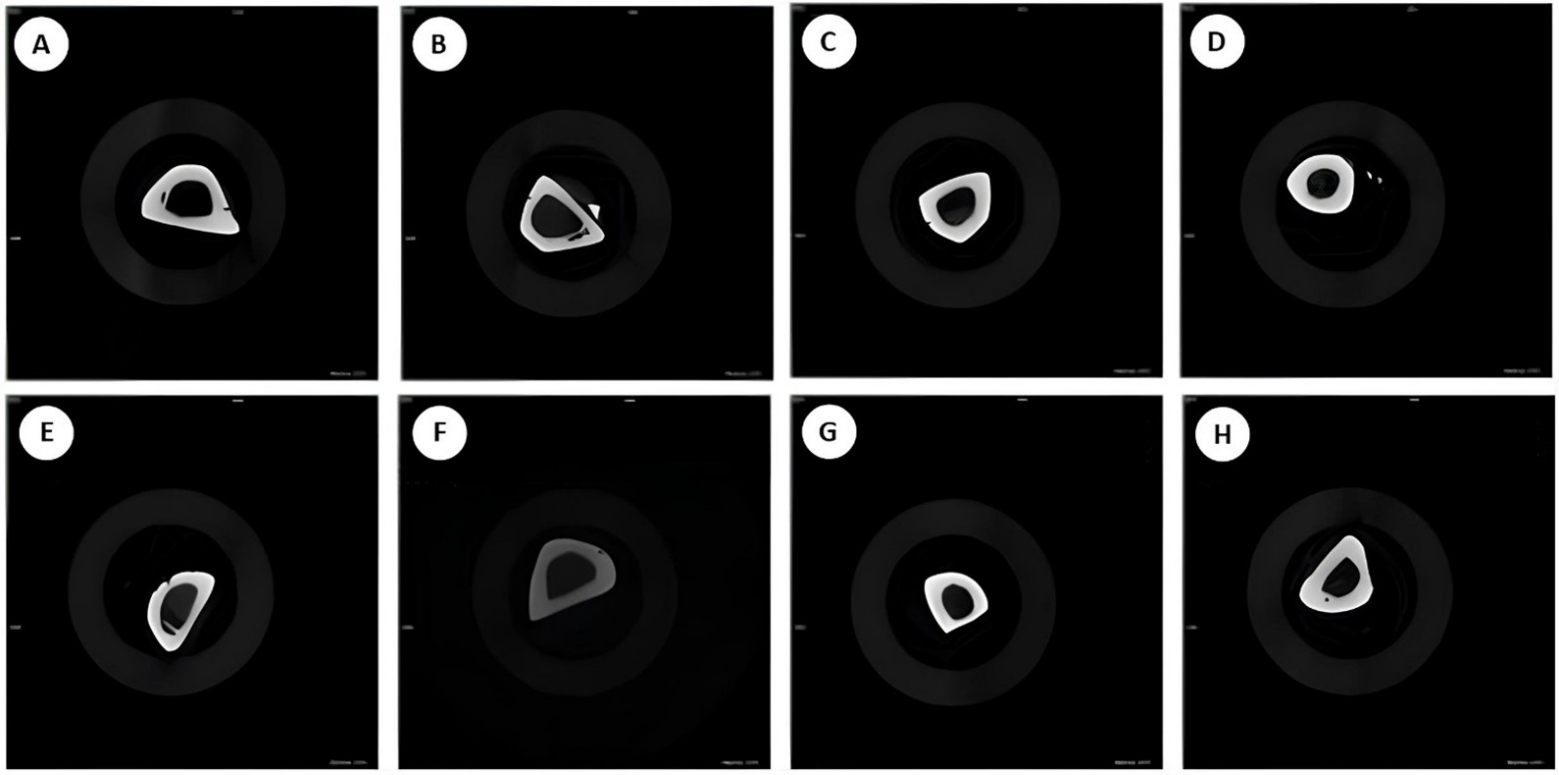
Computed Microtomography. Photomicrographs showing tibial characteristics in groups (A)SS 7d, (B)SS 28d (C), SS-Q10 7d, (D) SS-Q10 28d, (E)NIC 7d, (F) NIC 28d, (H) NIC-Q10 7d and (G) NIC-Q10 28d.

The means and standard deviation for each group and period are shown in Figs 12–14 and the photomicrographs in Fig 15. The intergroup analysis revealed that the SS group at 7 days had a larger bone area when compared to the NIC group at the same period. The SS-Q10 group at 28 days had a larger bone area when compared to the NIC group in both periods. The NIC-Q10 group at 28 days had a larger bone area when compared to the NIC groups at 7 and 28 days, NIC-Q10 at 7 days.

Regarding perimeter, there were no statistically significant differences between the groups in any period.

As for porosity and mineral density, the SS group at 7 days showed lower porosity and higher bone mineral density when compared to the NIC groups at 7 days and 28 days. The SS-Q10 group showed lower porosity and higher bone mineral density when compared to the NIC group in both periods. Finally, the NIC-Q10 group at 28 days had higher bone mineral density when compared to the NIC at 7 days and the NIC at 28 days and NIC-Q10 at 7 days, respectively.

## Discussion

As the worldwide population ages, the incidence of fractures increases [32]. Additionally, bone diseases and environmental factors, such as smoking [33], predispose to pathologic fractures of long bones. Accounting for the burden of fractures on patients and healthcare systems around the globe, it is worth searching for preventive therapies. CoQ10 is a natural coenzyme that exerts potent antioxidant and positive stimulatory effects on bone [20]. Animal studies are the first step for in vivo validation, essential for establishing cause and effect relationships. Considering that the pattern of bone loss in rats mimics the one observed in humans, the investigation of the impact of CoQ10 on the skeleton rats receiving NIC allows human extrapolations [3, 34].

The literature shows that supplementation for shorter periods, such as 7 days, regardless of the dose and supplementation, results in structural changes in the intestine of rats, including increased crypt depth [35]. For the longer period of 28 days, a study from 2001 demonstrated that supplementation with a certain substance was able to cause protective histomorphometric changes in estrogen-efficient rats [36]. Since supplementation can alter the microstructural parameters in long bones of osteoporotic rats, it is expected that these changes could be observed within the same period of time in our model. Hence, based on the recapitulation of optimal bone turnover and resistance to fracture by CoQ10, in the animals receiving NIC, interesting clinical inferences can be drawn by this study.

When evaluating structural parameters closely related to the occurrence of fractures (resistance and modulus of elasticity) [22–26], supplementation with CoQ10 improved all these parameters, thus indicating improvements to the bone quality and, thereby, resistance to fracture. However, CoQ10 didn’t induce significant changes to the macroscopic characteristics of the tibia (total area, perimeter, and porosity) of non-compromised animals (SS) animals, regardless of the period of analysis.

The negative impact of NIC on the bone was confirmed by significant changes to both macroscopic and structural parameters. Given that NIC upregulates osteoclastogenesis [22] and downregulates osteogenesis [4], it can be inferred that bone turnover is compromised by NIC. The imbalance between bone formation/bone resorption was confirmed by the micro-Ct, which revealed increased porosity and reduced density in group NIC. Owing to its regulation of oxidative stress and fibroblasts, osteoblast and osteoclast’s function [22–26], supplementation with CoQ10 exerted a protective effect on bone turnover in the presence of NIC, with increased density, reduced porosity, and resulted in greater resistance to fracture. Strikingly, although weight, width, length, and thickness (anatomical parameters) had been included to confirm that all animals were standardized before the experiments, groups NIC-Q10 exhibited a significant increase in these parameters compared to NIC. This finding suggests that CoQ10 might have affected growth in the presence of NIC.

Bone remodeling is a vastly studied process, in which even slight changes to the metabolism of cells involved in this phenomenon impact its overlapping cascade of events, ultimately altering the outcomes. NIC is known to jeopardize multiple phases related to bone remodeling [22]. This experiment confirmed the harmful impact of NIC on bone fracture parameters. Importantly, the capacity of CoQ10 in restoring bone homeostasis was also described. The benefits of CoQ10 on the evaluated parameters might be explained by four main mechanisms: its role in mitochondrial energy production, its potent antioxidant effects, its influence on collagen synthesis, and its role in the regulation of cellular apoptosis [37–41].

Regarding its role in mitochondrial energy production, it is important to highlight that CoQ10 is a relevant component of the electron transport chain, linking complex I to complex III, which results in the production of ATP through oxidative phosphorylation [27]. Osteoblasts require large amounts of ATP to sustain their functional activity; thus, the administration of CoQ10 promotes collagen biosynthesis in bone, while its deficiency can lead to decreased ATP production and, consequently, reduced osteoblastic activity [39]. In this context, the structural pattern observed in micro-CT shows that, despite the impairment caused by NIC, it can be inferred that animals supplemented with CoQ10 may have better maintained osteoblastic function.

Oxidative stress, characterized by the accumulation of reactive oxygen species (ROS), negatively impacts cell viability and osteoblast function and can be potentiated by NIC [40], as evidenced by the lower quality of bone tissue in animals that received this substance. CoQ10 acts as a fat-soluble antioxidant, regenerating the reduced form of ascorbic acid and other antioxidants such as vitamin E [41]. At the cellular level, it can reduce the oxidation of lipids and proteins, protecting cell membranes and maintaining the integrity of organelles. This effect is essential for reducing apoptosis in osteoblasts, which can be mediated by the inhibition of signaling pathways related to oxidative stress, such as the MAPK (mitogen-activated protein kinase) cascades and the NF-kB (nuclear factor kappa B). When activated, these pathways can lead to programmed cell death [29].

Still regarding the possible mechanisms by which CoQ10 enabled a protective action against NIC, it is possible to assume that CoQ10’s ability to modulate the expression of the gene that encodes collagen through transcription factors such as SP1 (Sp1 transcription factor) and AP-1 (Activator Protein-1) and its influence on the activation of AMP-active protein kinase (AMPK), which stimulates mitochondrial biogenesis and, consequently, the production of ATP, was able to improve the availability of energy necessary for collagen synthesis and bone mineralization, increasing the elasticity module and the resistance of the tibiae against fractures [42, 43].

The influence of CoQ10 on apoptosis and homeostasis helps explain how this substance acts not only as a protector against the deleterious effects of NIC but also as a positive modulator of the balance between bone formation and resorption. CoQ10 has the ability to inhibit apoptosis in osteoblasts, possibly by modulating the expression of anti-apoptotic proteins such as Bcl-2 (B-cell lymphoma 2) [44]. CoQ10 administration can lead to an increase in the levels of Bcl-2, which binds to pro-apoptotic proteins such as Bax (Bcl-2-associated X protein), preventing the formation of the apoptotic complex [44]. In osteoclasts, CoQ10 may also reduce the activation of the NF-kB signaling pathway, which is often triggered in response to oxidative stress, leading to an increase in bone resorption [45].

Although vastly studied, bone remodeling remains a complex phenomenon that depends on several factors, among which nutrient absorption reportedly plays an important role [21]. The gastrointestinal system is responsible for the digestion and absorption of a wide range of nutrients essential for maintaining bone integrity, and any dysfunction in this process can have significant implications for the entire skeletal system [19]. In experimental models, exposure to NIC has been associated with changes in the structure and function of intestinal villi, which are essential for the efficient absorption of nutrients [18].

There is an increasing body of evidence supporting that the intestine regulates numerous body functions and diseases [46–49]. Two main nutrients for bone homeostasis are directly affected by the absorption capacity of the intestine, calcium and vitamin D [21, 48]. Calcium is the most abundant mineral in the human body and an essential component of bone structure. Intestinal absorption of calcium is facilitated by the presence of vitamin D, which promotes the expression of calcium transport proteins in intestinal cells, thus increasing the efficiency of absorption of this mineral. Deficiencies in vitamin D can lead to a decrease in calcium absorption and, consequently, a decrease in bone mineral density, increasing the risk of osteoporosis and fractures [47–49].

Alarmingly, morphological changes to villi are attributed to smoking [50], suggesting that NIC alters bone turnover through indirect mechanisms. Intestinal villi are finger-like structures located in the mucous layer of the small intestine that expand the surface of this organ, optimizing the nutrient absorption process. The absorption capacity by the villi is directly related to its morphology [25, 33]. In this regard, it is worth highlighting that the crypts located in the basal layer of the mucosa favor the proliferation of enterocytic cells, which migrate to the villi and are essential for the efficient absorption of macronutrients and micronutrients. The efficient function of the crypts directly contributes to the intestine’s ability to process and absorb substances [51]. Although slightly, the supplementation with CoQ10 increased the size of the villi and the height of the intestinal crypts in the presence and absence of NIC. These changes may have contributed for achieving the improvements observed in the bone.

In addition to the nutrients mentioned, the integrity of the intestinal mucosa and the presence of a healthy microbiota have also been identified as factors that influence absorption and, consequently, bone health. Changes in the intestinal microbiota can affect the metabolism and absorption of nutrients, and it is evidenced in the literature that nicotine has modulating effects on this microbiota [52], which contributes to imbalances that can compromise bone health, as observed in the groups that received NIC. The impact of nicotine on the gut microbiota and its implications for nutrient absorption are still emerging areas of research, but early evidence suggests a significant relationship.

As previously described for the bone tissue, CoQ10 plays an important role in maintaining gastrointestinal health through its antioxidant action, neutralizing reactive oxygen species (ROS) that can cause oxidative damage to the epithelial cells of the gastrointestinal tract [44]. This action is vital for preserving the integrity of the mucosa and preventing dysbiosis and inflammation [53]. In addition, CoQ10’s role in ATP production provides the energy necessary for the proliferation and maintenance of intestinal epithelial cells, which are essential for nutrient absorption and immune defense [54]. CoQ10 also modulates the expression of cell adhesion proteins, such as occludins, which help to maintain the intestinal barrier and the integrity of the tight junctions between epithelial cells [55]. Finally, the literature indicate that CoQ10 can influence the activity of fibroblasts in the intestinal mucosa, promoting the healing and regeneration of lesions, which is essential for recovery from adverse gastrointestinal conditions [56].

The search for effective therapeutic/preventive modalities to improve any condition and/or the quality of life should account for the possible side effects, which means that risk-benefit measures ought never be neglected. Except for rare cases of gastrointestinal discomfort, CoQ10 is well tolerated, and no overdose or serious side effects are attributed to this substance, which supports its safety [57]. Importantly, there is no standard optimal dose for its use in multiple scenarios. Hence, we encourage further research to overcome this limitation.

Also, there are other conditions, such as diabetes, osteoporosis and radiotherapy, that influence the density of bones and increases the fracture incidence in worldwide population. Research is necessary to evaluate efficacy of CoQ10 in these conditions.

## Conclusion

Thus, within the limitations of the present study, it can be concluded that Coenzyme Q10 has strong potential to serve as an ally in the protection of the skeletal and gastrointestinal systems against nicotine exposure, since CoQ10 supplementation significantly increased fracture resistance, as evidenced by improvements in bone density, elastic modulus, and maximum strength, corroborating with existence literature. In addition, it increased nutrient absorption through intestinal protection, as indicated by improved intestinal crypt patterns. Future research should further investigate the long-term effects of CoQ10 on bone health in diverse populations, as well as explore its mechanisms of action to better understand its therapeutic potential.

## Supporting information

S1 File (PDF)
