# Supplementary material for: Effect of coenzyme Q10 on tibial fracture resistance in nicotine-exposed rats
Source: PLoS One. 2025 Jan 3;20(1):e0315462. doi: 10.1371/journal.pone.0315462 (PMC11698406; doi:10.1371/journal.pone.0315462)
Supplement: S1 File — (PDF) [file pone.0315462.s001.pdf]

Morphometric Analysis of the Tibias

	Experimental groups/ times							
	SS		SS-Q10		NIC		NIC-Q10	
	7d	28d	7d	28d	7d	28d	7d	28d
**Analysis**	**Mean and standard deviation**							
Width (mm)	4.53±0.31	4.57±0.17	4.43±0.2	4.53±0.3	3.30±0.23	4.82±0.35	4.46±0.23	4.51±0.25
Thickness (mm)	3.03±0.42	3.18±0.24	3.17±0.18	3.39±0.22	3.30±0.22	3.69±0.18	3.23±0.25	3.32±0.25
Length (mm)	41.80±2.76	44.32±2.01	43.59±1.41	43.37±0.92	41.63±1.27	42. 11±0.89	41.39±1.29	41.79±2.14
Weight (g)	1.06±0.19	1.08±0.12	1.11±0.9	1.11±0.15	1.16±0.15	1.07±0.09	1.07±0.07	0.95±0.09

Table showing the values (mean ± standard deviation) of the morphometric analysis (width, thickness, length and weight) of the tibias for each group and period. Source: from the authors themselves.

GRUOP	SAMPLE	WIDTH (mm)	THICKNESS (mm)	LENGTH (mm)	WEIGHT (g)
SS 7D	1	4,12	2,25	37,5	0,959
SS 7D	2	5	2,75	39,8	1,328
SS 7D	3	4,25	3,03	44	1,107
SS 7D	4	4,36	3,53	42,33	0,74
SS 7D	5	4,7	3,1	40,03	1,223
SS 7D	6	4,52	3,3	45,02	0,947
SS 7D	7	4,74	3,27	43,93	1,082
SS 28D	1	4,61	2,77	46,8	1,305
SS 28D	2	4,4	3,23	44,3	1,013
SS 28D	3	4,53	3,32	44,69	0,956
SS 28D	4	4,66	3,37	41,8	1,113
SS 28D	5	4,7	3,21	41,56	0,997
SS 28D	6	4,8	3,43	44,8	1,128
SS 28D	7	4,31	2,92	46,28	1,072
SS Q10 7D	1	4,35	3,18	40,7	1,125
SS Q10 7D	2	4,48	3,14	44,1	1,223
SS Q10 7D	3	4,08	2,97	42,9	1,111
SS Q10 7D	4	4,49	3,28	43,97	1,06
SS Q10 7D	5	4,76	3,5	44,85	0,967
SS Q10 7D	6	4,45	3,06	44,2	1,21
SS Q10 7D	7	4,41	3,07	44,42	1,053
SS Q10 28D	1	4,58	3,25	42,79	1,032
SS Q10 28D	2	4,6	3,17	42,04	1,343
SS Q10 28D	3	5,05	3,49	44,02	1,166
SS Q10 28D	4	4,45	3,78	44,78	0,953
SS Q10 28D	5	4,58	3,23	43,22	1,278
SS Q10 28D	6	4,05	3,3	43,89	1,001

SS Q10 28D	7	4,43	3,51	42,82	1,021
NIC 7D	1	5,03	3,31	42,59	1,317
NIC 7D	2	4,56	3,69	41,5	1,04
NIC 7D	3	4,68	3,43	41,15	1,351
NIC 7D	4	4,72	3,34	42,4	1,138
NIC 7D	5	4,24	3,13	40,72	1,232
NIC 7D	6	4,5	3,28	43,39	1,106
NIC 7D	7	4,19	2,95	39,65	0,937
NIC 28D	1	4,61	3,61	43	1,093
NIC 28D	2	5,23	3,41	42,56	0,901
NIC 28D	3	4,5	3,68	42,58	1,145
NIC 28D	4	5,09	4	41,65	1,058
NIC 28D	5	4,95	3,61	42,75	1,048
NIC 28D	6	4,31	3,78	40,41	1,19
NIC 28D	7	5,08	3,72	41,84	1,02
NIC Q10 7D	1	4,59	3,3	42,43	1,107
NIC Q10 7D	2	4,64	3,41	41,3	1,102
NIC Q10 7D	3	4,58	3,29	41,09	1,056
NIC Q10 7D	4	4,45	3,14	42,49	0,92
NIC Q10 7D	5	3,97	2,75	39,22	1,081
NIC Q10 7D	6	4,51	3,53	40,43	1,158
NIC Q10 7D	7	4,45	3,22	42,77	1,08
NIC Q10 28D	1	4,55	3,2	45,81	0,93
NIC Q10 28D	2	4,16	3,14	38,87	0,796
NIC Q10 28D	3	5,12	3,81	42,17	0,986
NIC Q10 28D	4	4,86	3,15	40,48	1,026
NIC Q10 28D	5	4,55	3,31	41,03	1,043
NIC Q10 28D	6	4,6	3,16	41,94	0,891
NIC Q10 28D	7	4,41	3,49	42,26	0,972

Mechanical Analysis of the Tibias

	**Experimental groups/ times**							
	**SS**		**SS-Q10**		**NIC**		**NIC-Q10**	
	**7d**	**28d**	**7d**	**28d**	**7d**	**28d**	**7d**	**28d**
**Analysis**	**Mean and standard deviation**							
Maximum tibial strength (N)	42.69±14.43	48.63±14.62	52.76±9.38	58.16±14.07	41.77±11.18	46.04±10.50	39.48±6.39	44.37±5.70
Maximum tibial resistance (N)	31.44±7.35 *	33.35±8.18 * α	47.96±5.26	43.86±3.98	28.31±3.85 *	22.65±3.84 *	35.93±3.45 α ¶	35.25±4.09 α ¶
Modulus of elasticity (Mpa)	300.45±84.18 *	270.22±70.49 *	582.27±117.95 ¶	498.52±102.17	230.42±55.19 * α	200.46±43.29 *¶	428.20±112.57 α	371.82±41.48

Table showing the values (mean ± standard deviation) of the mechanical analysis (maximum tibial strength, maximum tibial resistance and modulus of elasticity) of the tibias for each group and period. Statistical test: Shapiro-Wilk, Two-way ANOVA and Tukey test ($p \leq 0.05$). Source: from the authors themselves. **Legend for maximum tibial resistance analysis:** (*) statistically significant difference with the SS-Q10 group in both periods;(¶) statistically significant difference with the SS-Q10 group only at 7 days; (α) statistical difference with the NIC group at 28 days. **Legend for modulus of elasticity analysis:** (*) statistically significant difference with the SS-Q10 group in both periods; (¶) statistically significant difference with the NIC-Q10 group in both periods; (α) statistical difference between groups in the same period.

GROUP	SAMPLE	MAXIMUM TIBIAL STRENGTH (N)	MAXIMUM TIBIAL RESISTANCE (N)	MODULUS OF ELASTICITY (MPA)
SS 7D	1	21,08	37,91	240,86
SS 7D	2	57,81	57,33	938,26
SS 7D	3	52,37	50,33	556,49

SS 7D	4	39,45	27,73	275,23
SS 7D	5	25,44	21,12	160,54
SS 7D	6	50,53	38,5	436,35
SS 7D	7	52,16	38,19	340,28
SS 28D	1	38,9	41,24	309,87
SS 28D	2	36,18	29,96	258,41
SS 28D	3	56,93	42,75	179,45
SS 28D	4	33,53	23,76	193,83
SS 28D	5	41,49	32,12	256,77
SS 28D	6	70,73	46,97	379,58
SS 28D	7	62,64	63,92	617,36
SS Q10 7D	1	54,75	46,67	490,88
SS Q10 7D	2	61,75	52,43	515,06
SS Q10 7D	3	35,23	36,71	480,03
SS Q10 7D	4	53,46	41,5	388,19
SS Q10 7D	5	49,72	31,97	358,96
SS Q10 7D	6	63,79	57,41	745,21
SS Q10 7D	7	50,6	45,65	756,76
SS Q10 28D	1	64,27	49,82	283,37
SS Q10 28D	2	52,16	41,42	516,2
SS Q10 28D	3	79,98	48,76	480,17
SS Q10 28D	4	69,58	41,03	222,87
SS Q10 28D	5	51,89	39,41	367,11
SS Q10 28D	6	51,76	44,01	715,82
SS Q10 28D	7	37,47	25,75	234,06
NIC 7D	1	31,76	21,61	127,01
NIC 7D	2	50,6	30,56	318,71

NIC 7D	3	38,7	26,36	214,09
NIC 7D	4	49,72	35,41	360,64
NIC 7D	5	57,81	34,14	349,39
NIC 7D	6	36,73	28,45	211,23
NIC 7D	7	27,07	27,84	304,36
NIC 28D	1	51,42	32,09	237,28
NIC 28D	2	43,05	26,55	386,81
NIC 28D	3	57,67	35,49	336,59
NIC 28D	4	43,8	20,17	146,07
NIC 28D	5	58,35	33,92	327,39
NIC 28D	6	29,45	16,18	157,56
NIC 28D	7	38,56	20,57	163,74
NIC Q10 7D	1	37,95	28,47	275,26
NIC Q10 7D	2	48,9	33,99	352,96
NIC Q10 7D	3	37,61	28,45	318,09
NIC Q10 7D	4	39,58	33,83	401,78
NIC Q10 7D	5	34,14	42,6	656,79
NIC Q10 7D	6	31,29	20,88	171,67
NIC Q10 7D	7	46,86	38,09	478,81
NIC Q10 28D	1	40,53	32,62	383,64
NIC Q10 28D	2	39,17	35,82	298,35
NIC Q10 28D	3	46,32	23,37	245,34
NIC Q10 28D	4	56,04	43,58	427,92
NIC Q10 28D	5	41,01	30,85	272,48
NIC Q10 28D	6	42,92	35,04	424,68
NIC Q10 28D	7	44,62	31,15	246,64

Analysis of Computerized Microtomography (Micro Ct) and Densitometry tibial bone

	Experimental groups/ times							
	SS		SS-Q10		NIC		NIC-Q10	
	7d	28d	7d	28d	7d	28d	7d	28d
**Analysis**	**Mean and standard deviation**							
Total bone area (%)	45.19±6.66 *	47.17±6.58	47.94±12.60	46.19±7.90 *¶	35.35±7.08 α	35.87±7.40	37.62±7.78	45.88±2.83 *¶
Perimeter (mm)	19.84±1.53	20.57±3.87	21.58±13.17	19.20±2.82	19.25±2.7	19.11±2.37	18.56±3.05	19.62±1.60
Porosity and bone mineral density (%)	53.55±6.60 *	55.35±7.81	53.88±7.89 *	54.53±6.64 *	65.30±7.74	64.91±5.04	62.48±7.78	54.21±2.82 *¶

Table showing the values (mean ± standard deviation) of the analysis of Computerized Microtomography (Micro Ct) and densitometry tibial bone for each group and period. Statistical test: Shapiro-Wilk, Two-way ANOVA and Tukey test ($p \leq 0.05$). Source: from the authors themselves. **Legend for total bone area analysis:** (*) statistically significant difference with the NIC group at 7 days; (¶) statistically significant difference with the NIC group at 28 days; (α) statistically significant difference with the NIC-Q10 group at 28 days. **Legend for porosity and bone mineral density analysis:** (*) statistically significant difference with the NIC group in both periods; (¶) statistically significant difference with the NIC-Q10 group at 7 days.

GROUP	SAMPLE	TOTAL BONE AREA (%)	PERIMETER (mm)	POROSITY AND BONE MINERAL DENSITY (%)
SS 7D	1	37,6	17,97	47,42
SS 7D	2	38,66	18,76	60,43
SS 7D	3	47,28	19,73	52,23
SS 7D	4	40,49	17,78	48,07
SS 7D	5	57,48	21,52	44,49
SS 7D	6	44,69	21,45	63,14
SS 7D	7	49,93	21,34	59,1
SS 28D	1	33,4	21,16	57,72
SS 28D	2	55,4	20,11	44,89
SS 28D	3	51,54	22,08	52,82
SS 28D	4	51,3	13,8	60,71
SS 28D	5	48,78	18,5	54,35
SS 28D	6	46,98	20,11	68,31

SS 28D	7	42,9	28,23	48,69
SS Q10 7D	1	53,75	19,17	39,48
SS Q10 7D	2	45,7	10,05	59,25
SS Q10 7D	3	53,8	18,21	59,16
SS Q10 7D	4	24,93	18,14	49,35
SS Q10 7D	5	48,46	23,12	64,52
SS Q10 7D	6	72,6	10,26	50,2
SS Q10 7D	7	36,34	18,38	55,23
SS Q10 28D	1	41,56	19,71	63,12
SS Q10 28D	2	32,1	20,78	52,54
SS Q10 28D	3	43,23	22,87	49,19
SS Q10 28D	4	48,12	17,93	51,21
SS Q10 28D	5	59,87	14,84	59,29
SS Q10 28D	6	51,34	19,95	62,24
SS Q10 28D	7	47,16	15,94	44,12
NIC 7D	1	48,16	23,96	72,86
NIC 7D	2	33,78	19,54	73,64
NIC 7D	3	31,6	18,28	54,94
NIC 7D	4	25,61	19,61	53,66
NIC 7D	5	32,75	21,27	59,63
NIC 7D	6	32,4	16,21	68,56
NIC 7D	7	43,18	26,14	59,85
NIC 28D	1	22,55	33,43	71,11
NIC 28D	2	36,96	32,23	56,84
NIC 28D	3	42,15	42,92	72,63
NIC 28D	4	30,79	49,92	62,59
NIC 28D	5	39,07	49,17	65,31
NIC 28D	6	34,42	36,13	63,45
NIC 28D	7	45,21	31,18	62,46
NIC Q10 7D	1	30,6	21,7	53,55
NIC Q10 7D	2	45,16	21,3	54,96
NIC Q10 7D	3	28,12	14,21	55,35
NIC Q10 7D	4	39,11	17,11	63,25
NIC Q10 7D	5	40,42	14,62	64,15
NIC Q10 7D	6	49,95	22,31	72,76
NIC Q10 7D	7	29,53	18,61	73,35
NIC Q10 28D	1	48,84	19,77	51,45
NIC Q10 28D	2	41,85	17,1	53,91
NIC Q10 28D	3	47,71	18,93	53,94
NIC Q10 28D	4	44,70	19,43	52,75
NIC Q10 28D	5	41,94	19,62	51,89
NIC Q10 28D	6	47,85	19,4	56,9
NIC Q10 28D	7	48,33	23,12	58,65

Histometric Analysis of the Intestines

LENGTH OF INTESTINAL VILLI	NIC 28D	NIC-Q10 28D	SS 28D	SS-Q10 28D
1	130,2	101,54	108,33	113,49
2	95,34	98,74	130,05	124,76
3	117,38	179,71	129,18	107,92
4	119,97	150,43	106	123,6
5	105,41	123,45	115,36	106,46
6	120,06	124,84	115,39	145,35
7	96,28	126,19	118,88	112,93
8	127,96	101,26	136,58	158,23
**MEAN**	**114,08**	**125,77**	**119,97**	**124,09**
**SD**	**13,51**	**27,88**	**10,93**	**18,62**

HEIGHT OF INTESTINAL CRYPTS	NIC 28D	NIC-Q10 28D	SS 28D	SS-Q10 28D
1	19,87	21,42	49,8	23,99
2	23,37	22,18	32,25	31,28
3	25,77	25,3	39,56	75,40
4	24,67	39,44	17,43	48,91
5	24,89	45,21	21,33	37,73
6	23,43	24,15	8,75	29,64
7	37,09	23,4	23,53	44,61
8	28,76	28,26	19,9	43,90
**MEAN**	**24,07**	**30,38**	**31,32**	**44,15**
**SD**	**1,90**	**7,79**	**9,30**	**14,20**
